# Hyperbaric oxygen therapy improves neurocognitive functions and symptoms of post-COVID condition: randomized controlled trial

**DOI:** 10.1038/s41598-022-15565-0

**Published:** 2022-07-12

**Authors:** Shani Zilberman-Itskovich, Merav Catalogna, Efrat Sasson, Karin Elman-Shina, Amir Hadanny, Erez Lang, Shachar Finci, Nir Polak, Gregory Fishlev, Calanit Korin, Ran Shorer, Yoav Parag, Marina Sova, Shai Efrati

**Affiliations:** 1 Sagol Center for Hyperbaric Medicine and Research, Shamir (Assaf Harofeh) Medical Center, Zerifin, Israel; 2 Sackler School of Medicine, Tel- Aviv University, Tel-Aviv, Israel; 3 Sagol School of Neuroscience, Tel-Aviv University, Tel-Aviv, Israel

**Keywords:** Infectious diseases, Neurological disorders

## Abstract

Post-COVID-19 condition refers to a range of persisting physical, neurocognitive, and neuropsychological symptoms after SARS-CoV-2 infection. The mechanism can be related to brain tissue pathology caused by virus invasion or indirectly by neuroinflammation and hypercoagulability. This randomized, sham-control, double blind trial evaluated the effect of hyperbaric oxygen therapy (HBOT or HBO2 therapy) on post-COVID-19 patients with ongoing symptoms for at least 3 months after confirmed infection. Seventy-three patients were randomized to receive daily 40 session of HBOT (n = 37) or sham (n = 36). Follow-up assessments were performed at baseline and 1–3 weeks after the last treatment session. Following HBOT, there was a significant group-by-time interaction in global cognitive function, attention and executive function (d = 0.495, p = 0.038; d = 0.477, p = 0.04 and d = 0.463, p = 0.05 respectively). Significant improvement was also demonstrated in the energy domain (d = 0.522, p = 0.029), sleep (d = − 0.48, p = 0.042), psychiatric symptoms (d = 0.636, p = 0.008), and pain interference (d = 0.737, p = 0.001). Clinical outcomes were associated with significant improvement in brain MRI perfusion and microstructural changes in the supramarginal gyrus, left supplementary motor area, right insula, left frontal precentral gyrus, right middle frontal gyrus, and superior corona radiate. These results indicate that HBOT can induce neuroplasticity and improve cognitive, psychiatric, fatigue, sleep and pain symptoms of patients suffering from post-COVID-19 condition. HBOT’s beneficial effect may be attributed to increased brain perfusion and neuroplasticity in regions associated with cognitive and emotional roles.

## Introduction

As of January 2022, the severe acute respiratory syndrome coronavirus 2 (SARS-CoV-2) pandemic has resulted in more than 300 million infected cases. Even though most infected patients recover, 10–30% remain with persistent symptoms that have devastating effects on their quality of life^1,2^. The World Health Organization has recognized this clinical condition and defined it as post-COVID-19 condition. This condition is confirmed three months from the onset of COVID-19 with having physical, neurocognitive and psychiatric symptoms that persist for more than two months and cannot be explained by an alternative diagnosis^1^. Neurocognitive and psychiatric symptoms include decreased executive functions, anxiety, depression and posttraumatic stress symptoms^3,4^. Most common physical symptoms include fatigue, dyspnea, ageusia, anosmia, insomnia, headaches and systemic widespread pain^5^.

The pathogenesis of post-COVID-19 condition is not yet determined. Suggested mechanisms include direct brain invasion of the virus, dysregulated immunologic responses, thrombotic disease, mitochondrial dysfunction and vascular injury with secondary tissue hypoxia^6,7^. Currently studied treatment options of post-COVID-19 condition are targeted anti-inflammatory molecules, specific diets, and cognitive behavioral therapy. However, none have been determined effective^8–10^.

In recent years, evidence has been accumulated about the neuroplasticity effects of hyperbaric oxygen therapy (HBOT)^11–19^. It is now realized, that the combined action of hyperoxia and hyperbaric pressure, leads to significant improvement in tissue oxygenation while targeting both oxygen and pressure sensitive genes^11^. Preclinical and clinical studies have demonstrated several neuroplasticity effects including anti-inflammatory, mitochondrial function restoration, increased perfusion via angiogenesis and induction of proliferation and migration of stem cells^11–13,20,21^. Robbins et al. suggested a possible benefit with HBOT in a recent case series of ten post-COVID-19 condition patients^22^.

The aim of the current study was to evaluate the effects of HBOT on patients suffering from post-COVID-19 condition, with ongoing symptoms for at least 3 months after confirmed infection, in a randomized, sham-control, double blind clinical trial.

## Results

### Patient characteristics and randomization

Ninety-one patients were eligible to participate in the study. Twelve patients did not complete baseline evaluation. Seventy-nine were randomized to one of the two arms. Two patients from the control group withdrew their consent during treatment, and one patient was excluded due to poor compliance and did not complete the assessments. Two patients from the HBOT group were excluded, one due to intercurrent illness, and one due to a personal event that prevented completion of the protocol. An additional patient from the HBOT group withdrew his consent during treatment. Accordingly, 37 patients from the HBOT group and 36 patients from the control group completed the protocol and were included in the analysis. The patient flowchart and study timeline are presented in Supplementary Fig. 1. Patient baseline characteristics are detailed in Table 1. No statistically significant differences between the two groups were observed in baseline characteristics. Post-COVID-19 self-reported symptoms data are provided in Supplementary Tables 1–2. No significant differences were observed in baseline symptoms between the two groups.

**Table 1:** Baseline characteristics.

	HBOT	Control	p-value
N	37	36	
Age (years)	48.4 ± 10.6	47.8 ± 8.5	0.784
Males	18 (48.6)	11 (30.6)	0.153
Female	19 (51.4)	25 (69.4)	0.153
BMI (Kg/m^2^)	26.9 ± 5.1	26.5 ± 4.7	0.690
Years of education	14.6 ± 2.7	15.1 ± 3.6	0.592
**Marital status**			
Single	5 (13.5)	7 (19.4)	0.543
Married	27 (73.0)	22 (61.1)	0.326
Divorced	3 (8.1)	6 (16.7)	0.308
Widowed	2 (5.4)	1 (2.8)	1.000
Number of children	2.5 ± 1.4	2.4 ± 1.5	0.839
**Employment status**			
Full time	24 (64.9)	22 (61.1)	0.811
Part time	9 (24.3)	11 (30.6)	0.607
Not employed	4 (10.8)	3 (8.3)	1.000
Time from infection (days)	159.1 ± 71.3	171.5 ± 66.4	0.450
Hospitalized*	4 (10.8)	8 (22.2)	0.221
MoCA—cognitive assessment	25.4 ± 3.6	25.0 ± 3.3	0.601
**High risk conditions**			
BMI^†^ > 30	11 (29.7)	9 (25.0)	0.794
Age > 60 years	4 (10.8)	4 (11.1)	1.000
Cancer	0 (0.0)	0 (0.0)	1.000
Diabetes mellitus	1 (2.7)	1 (2.8)	1.000
Hypertension	4 (10.8)	2 (5.6)	0.674
Heart disease	1 (2.7)	1 (2.8)	1.000
Immune deficiency	0 (0.0)	0 (0.0)	1.000
Asthma	2 (5.4)	1 (2.8)	1.000
Other chronic lung diseases	0 (0.0)	0 (0.0)	1.000
Chronic liver disease	0 (0.0)	4 (11.1)	0.054
Chronic kidney disease	0 (0.0)	0 (0.0)	1.000
Hematologic disease\disorder	0 (0.0)	0 (0.0)	1.000
Chronic neurological impairment\disease	1 (2.7)	1 (2.8)	1.000
**Smoking**			
Current	0 (0.0)	0 (0.0)	1.000
Previous	10 (27.0)	7 (19.4)	0.581

Data presented as n (%); continuous data, mean ± SD; ^†^The body-mass index is the weight in kilograms divided by the square of the height in meters. *During COVID-19 infection. *MoCA* Montreal Cognitive Assessment.

Participants’ blinding was found to be reliable, where the correct group allocation perception rate was 54.1% and 66.7% (p = 0.271) in the HBOT and control groups respectively (Supplementary Fig. 2).

### Primary outcome

There were no significant differences between the groups in all baseline cognitive domains. There was a significant group-by-time interaction in the global cognitive score post-HBOT compared to the control group, with a medium net effect size (d = 0.495, p = 0.038). Both attention and executive function domains had significant group-by-time interactions (d = 0.477, p = 0.04 and d = 0.463, p = 0.05 respectively) (Table 2, and Supplementary Table 3).

**Table 2:** Neurocognitive performance changes.

	HBOT				Control				p-value baseline	Net effect size*	ANOVA (group-by-time) interaction	
	Pre	Post	p-value**	Change	Pre	Post	p-value**	Change			F	p-value
N	37				36							
Score	98.3 ± 11.1	104.1 ± 7.2	**0.0001**	5.8 ± 7.9	98.9 ± 8.5	101.3 ± 8.9	0.0105	2.4 ± 5.4	0.821	0.495	4.469	0.038
Memory	93.7 ± 13.4	102.0 ± 10.9	**0.0001**	8.3 ± 11.2	94.9 ± 12.2	102.1 ± 8.7	**0.0000**	7.2 ± 8.5	0.695	0.111	0.226	0.636
Executive function	103.5 ± 13.1	109.0 ± 8.2	**0.0029**	5.6 ± 10.6	102.5 ± 10.3	103.8 ± 10.5	0.2526	1.3 ± 6.8	0.725	0.477	4.159	0.045
Attention	97.3 ± 16.0	101.9 ± 9.0	0.0292	4.6 ± 12.4	99.6 ± 8.2	99.4 ± 10.1	0.8495	− 0.3 ± 8.3	0.434	0.463	3.914	0.052
Information processing speed	94.8 ± 14.2	102.4 ± 13.0	0.**0003**	7.6 ± 11.4	94.4 ± 14.2	98.3 ± 17.7	0.0734	3.9 ± 12.7	0.910	0.303	1.673	0.200
Motor skills	102.4 ± 12.6	105.3 ± 8.3	0.0827	2.9 ± 10.0	102.9 ± 8.4	102.9 ± 9.0	0.9639	0.1 ± 6.7	0.858	0.338	2.079	0.154

Data are presented as mean ± SD; Bold, significant after Bonferroni correction; * Cohen's d net effect size; ** pre-post treatment/ sham P-value. The follow up assessments were performed 1–3 weeks after the last treatment session.

### Secondary outcomes

**Figure 1:**
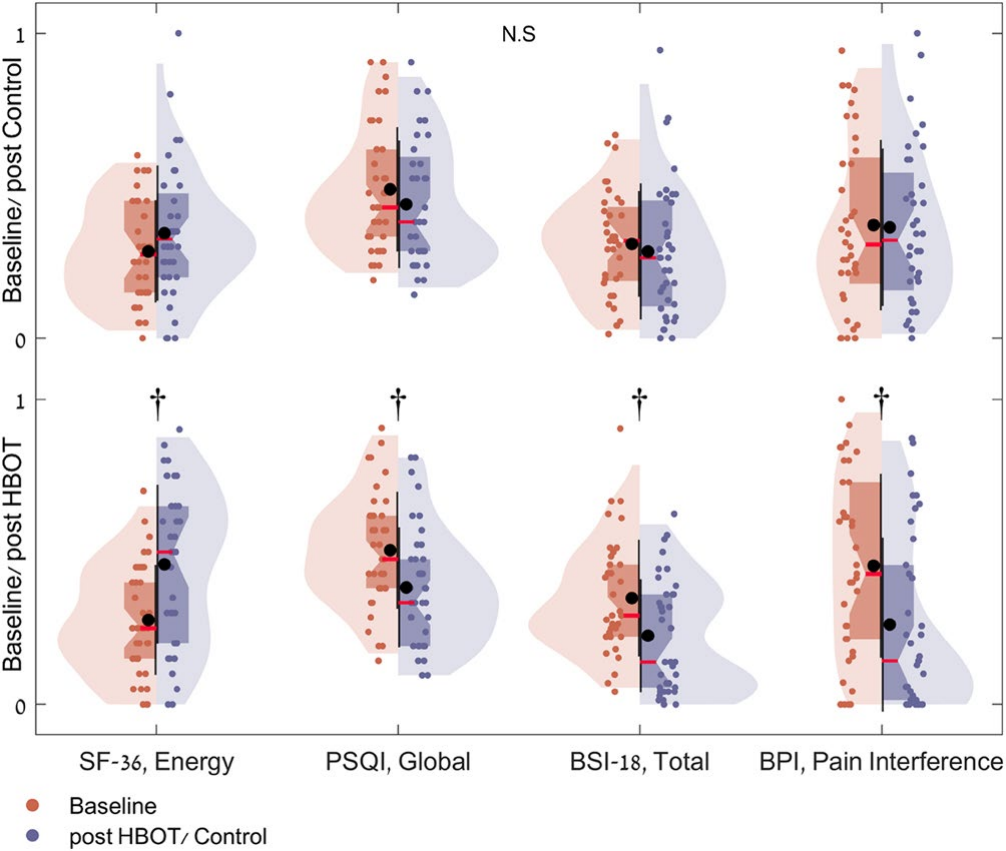
Questionnaire results analysis shown in violin plots of actual distribution, and in boxplots. Values are normalized to answer scale range: SF-36, Energy [0..100], PSQI, Global [0..21], BSI-18, Total [0..72] and BPI, Pain Interference [0..10]. The red mark indicates the median, and the bottom and top edges of the box indicate the 25th and 75th percentiles, respectively. Black marks indicate mean and standard deviation. ^†^p < 0.0001, *N.S.* not significant (see also Table 3).

Questionnaire analysis is summarized in Fig. 1, Table 3, and Supplementary Table 4. At baseline, there were no significant differences in all domains between the groups. In the SF-36*,* the HBOT group improved in both physical limitation and energy with group-by-time significant interactions of (d = 0.544, p = 0.023) and (d = 0.522 p = 0.029). In the PSQI, the HBOT group improved in the global sleep score with a significant group-by-time interaction (d = − 0.48, p = 0.042). Improvements in psychological symptoms were also demonstrated after HBOT with significant group-by-time interaction and large effect size in the total BSI-18 score (d = 0.636, p = 0.008). Both somatization (d = 0.588, p = 0.014) and depression (d = 0.491, p = 0.04) scores showed significant group-by-time interactions. The anxiety score improved significantly in the HBOT and did not change in the control group. However, the group-by-time interaction did not reach significance level (p = 0.079). Post-HBOT improvement was also found in the BPI pain interface score with a significant group-by-time interaction and a large effect size (d = 0.737, p = 0.001).

**Table 3:** Questionnaire results analysis.

	HBOT				Control				p-value baseline	Net effect size*	ANOVA (group-by-time) interaction	
	Pre	Post	p-value**	Change	Pre	Post	p-value**	Change			F	p-value
N	37				36							
**SF-36**												
Physical functioning	60.3 ± 24.7	63.0 ± 29.3	0.439	2.7 ± 21.0	50.7 ± 24.4	58.6 ± 26.9	0.010	7.9 ± 17.5	0.105	− 0.269	1.322	0.254
Physical limitations	16.9 ± 26.0	50.7 ± 38.3	**0.000**	33.8 ± 40.9	29.2 ± 34.1	38.9 ± 38.8	0.224	9.7 ± 47.2	0.092	0.546	5.43	0.023
Emotional limitations	33.3 ± 33.8	60.4 ± 37.8	**0.001**	27.0 ± 42.9	32.4 ± 37.3	50.0 ± 43.4	0.024	17.6 ± 44.7	0.913	0.215	0.846	0.361
Energy	27.7 ± 17.8	45.9 ± 25.7	**0.000**	18.2 ± 24.4	28.5 ± 16.5	34.4 ± 21.9	0.121	6.0 ± 22.5	0.851	0.522	4.976	0.029
Emotional wellbeing	49.9 ± 20.1	64.0 ± 21.5	**0.000**	14.1 ± 17.8	51.2 ± 18.7	55.3 ± 22.7	0.332	4.1 ± 25.1	0.783	0.459	3.841	0.054
Social function	47.6 ± 25.5	67.6 ± 25.7	**0.000**	19.9 ± 25.6	51.4 ± 26.3	61.5 ± 26.9	0.020	10.1 ± 24.8	0.543	0.391	2.795	0.099
Pain domain	39.4 ± 33.6	59.5 ± 33.0	**0.000**	20.1 ± 28.6	41.1 ± 30.2	54.2 ± 28.4	**0.007**	13.1 ± 27.1	0.821	0.254	1.179	0.281
General health domain	51.5 ± 18.3	60.9 ± 20.4	**0.003**	9.5 ± 18.2	46.5 ± 13.5	49.4 ± 18.6	0.397	2.9 ± 20.4	0.200	0.338	2.088	0.153
**PSQI**												
Global	10.6 ± 4.0	8.1 ± 4.1	**0.000**	− 2.6 ± 3.1	10.3 ± 4.2	9.2 ± 4.3	0.068	− 1.0 ± 3.3	0.704	− 0.486	4.302	0.042
Sleep quality	2.1 ± 0.8	1.5 ± 0.9	**0.001**	− 0.6 ± 1.0	2.1 ± 0.7	1.8 ± 0.8	0.014	− 0.3 ± 0.7	0.990	− 0.310	1.753	0.19
Sleep latency	1.9 ± 1.1	1.3 ± 1.2	**0.000**	− 0.6 ± 0.8	1.9 ± 1.0	1.6 ± 1.1	0.012	− 0.3 ± 0.8	0.837	− 0.308	1.73	0.193
Sleep duration	1.5 ± 1.1	1.4 ± 0.9	0.500	− 0.1 ± 1.0	1.3 ± 1.0	1.6 ± 0.9	0.133	0.2 ± 0.9	0.457	− 0.360	2.364	0.129
Sleep efficiency	0.5 ± 0.9	0.4 ± 0.8	0.096	− 0.1 ± 0.5	0.5 ± 1.0	0.4 ± 0.6	0.226	− 0.2 ± 0.8	0.849	0.047	0.041	0.840
Sleep disturbances	1.9 ± 0.6	1.5 ± 0.6	**0.001**	− 0.4 ± 0.6	1.7 ± 0.6	1.6 ± 0.6	0.291	− 0.1 ± 0.6	0.224	− 0.465	3.94	0.051
Sleep medication	0.8 ± 1.2	0.5 ± 1.1	0.134	− 0.3 ± 1.1	0.7 ± 1.1	0.6 ± 0.9	0.845	− 0.0 ± 0.8	0.740	− 0.251	1.15	0.287
Daytime dysfunction	2.0 ± 0.7	1.5 ± 0.9	**0.004**	− 0.5 ± 1.0	2.0 ± 0.8	1.7 ± 0.8	0.039	− 0.3 ± 0.9	0.882	− 0.221	0.891	0.348
**BSI-18**												
Total	25.1 ± 13.6	16.2 ± 13.2	**0.000**	− 8.9 ± 10.6	22.3 ± 12.3	20.5 ± 15.8	0.362	− 1.8 ± 11.7	0.362	− 0.636	7.372	**0.008**
Somatization	9.3 ± 6.0	6.2 ± 5.9	**0.000**	− 3.1 ± 3.8	8.3 ± 4.5	7.7 ± 5.5	0.531	− 0.5 ± 5.0	0.397	− 0.588	6.312	0.014
Depression	7.4 ± 6.1	4.1 ± 4.7	**0.001**	− 3.2 ± 5.4	6.3 ± 5.1	5.6 ± 6.3	0.300	− 0.8 ± 4.4	0.446	− 0.491	4.395	0.04
Anxiety	8.4 ± 4.6	5.9 ± 4.7	**0.002**	− 2.5 ± 4.5	7.7 ± 5.3	7.2 ± 6.3	0.571	− 0.5 ± 5.3	0.534	− 0.417	3.169	0.079
**BPI**												
Pain severity score	1.6 ± 2.3	1.4 ± 2.5	0.520	− 0.2 ± 1.8	1.5 ± 1.7	1.3 ± 2.2	0.721	− 0.1 ± 2.3	0.701	− 0.024	0.011	0.917
Pain interference score	4.5 ± 3.0	2.6 ± 2.8	**0.000**	− 1.9 ± 2.3	3.7 ± 2.8	3.6 ± 2.5	0.855	− 0.1 ± 2.5	0.223	− 0.784	11.204	**0.001**

Data are presented as mean ± SD; Bold, significant after Bonferroni correction; *Cohen's d net effect size; **Pre-post treatment/sham p-value. The follow up assessments were performed 1–3 weeks after the last treatment session.

#### Brain perfusion

**Figure 2:**
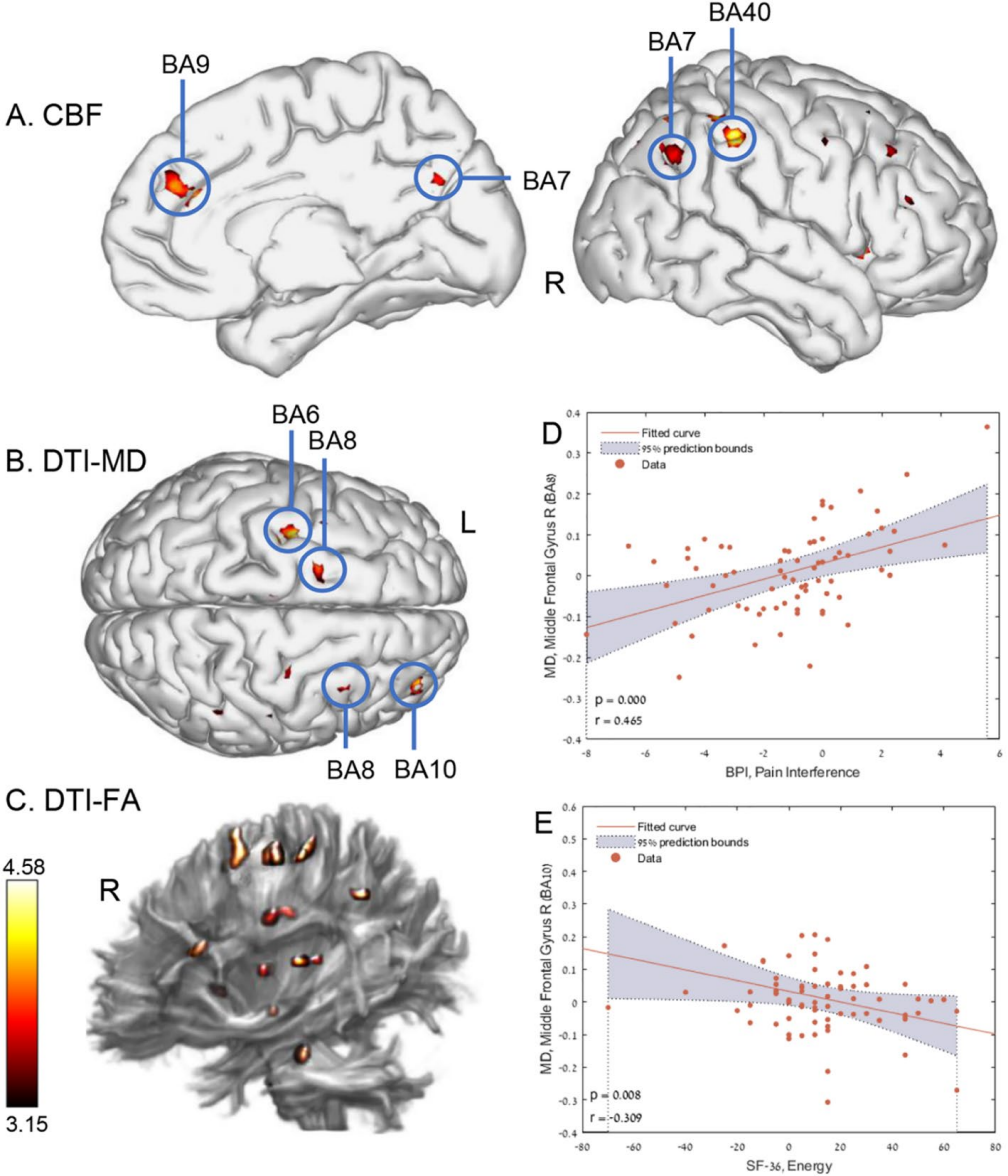
Brain regions with significant post-hyperbaric oxygen therapy changes compared to control. Group-by-time interaction ANOVA model in: (**A**) cerebral blood flow (CBF) in GM, p < 0.0005, uncorrected, (**B**) mean diffusivity DTI-MD in GM, p < 0.002, uncorrected, (**C**) fractional anisotropy DTI-FA in WM, p < 0.002, uncorrected. (**D**) significant correlation between pain interference score and the right middle formal gyrus MD (BA8). (**E**) significant correlation between the energy score and the right middle frontal gyrus MD (BA10). r is Pearson's correlation coefficient. The 95% prediction interval is presented in the shaded area. *CBF* cerebral blood flow, *MD* mean diffusivity, *FA* fractional anisotropy, *GM* gray matter, *WM* white matter, *R* right, *L* left, *BA* Brodmann area. (**A**) and (**B**) brain images were created using BrainNet Viewer software (http://www.nitrc.org/projects/bnv/)^43^. (**C**) Brain image was created using ExploreDTI software (https://www.exploredti.com/)^44^.

One patient was excluded due to excessive head motion. Therefore a total of 36 patients from each group were analyzed. Voxel-based analysis revealed significant gray-matter CBF increases in the HBOT group compared to the controls as shown in Fig. 2A, and Supplementary Table 5. Significant group-by-time interactions were demonstrated in the left and right supramarginal gyrus (BA40), left anterior cingulate gyrus (BA10/BA32), right superior parietal lobule (BA7), left supplementary motor area (BA6), left parahippocampal gyrus, and the right insula (BA13).

#### Brain microstructure

Voxel-based DTI analysis of brain gray-matter mean diffusivity (MD) maps is shown in Fig. 2B and Supplementary Table 6. Significant group-by-time interactions were demonstrated in the left frontal precentral gyrus (BA6), and the right middle frontal gyrus (BA10, BA8).

Voxel-based DTI analysis of brain white-matter fractional anisotropy (FA) maps is shown in Fig. 2C, and Supplementary Table 7. Significant group-by-time interactions were demonstrated in both right and left superior corona radiata.

There were significant correlations between pain interference and energy scores and MD changes in the right middle frontal gyrus (r = 0.465, p < 0.0001, r = − 0.309, p = 0.008 respectively). The NeturTrax global score correlated to increased perfusion in the left supramarginal gyrus (r = 0.285, p = 0.0152) (Fig. 2D,E).

The results of the smell and taste evaluations are summarized in Supplementary Table 8, and Supplementary Figs. 3–4. Impairment in odor detection at baseline was found in 27(73%) of the HBOT patients and in 25(69%) of the control. Both groups’ odor detection improved significantly and there was no significant group-by-time interaction.

Abnormal taste sensation at baseline was found in 18(49%) patients from the HBOT group and in 12(33%) from the control. Compared to baseline, there were significant improvements in the HBOT group in the total taste score, and in sweet and bitter taste domains (p = 0.003, 0.007 and 0.014 respectfully). In the control group, there was a significant improvement in only the sweet domain (p = 0.034). However, there were no significant group-by-time interactions.

Baseline blood tests, and pulmonary function tests were within the normal range. No significant changes were observed post-treatment (Supplementary Tables 9–10).

### Safety

The reported side effects are present in Supplementary Table 11. There was no significant difference in any of the reported side effect between the groups (35.1% and 38.9%, p = 0.739 in the HBOT and control groups respectively). None of the patients needed to discontinue the treatment because of side effects.

## Discussion

This is the first prospective, randomized sham-controlled trial demonstrating significant improvement beyond the expected clinical recovery course of post-COVID-19 condition. We found that HBOT improves dysexecutive functions, psychiatric symptoms (depression, anxiety and somatization), pain interference symptoms and fatigue. Those changes were associated with increased CBF and brain microstructural changes in frontal, parietal and limbic regions associated with cognitive and psychiatric roles.

Becker et al. show that the main cognitive impairments in post-COVID-19 condition is dysexecutive, or brain fog, with considerable implications for occupational, psychological, and functional outcomes^23^. In this study, improvements in the memory domain was in both groups, which can be attributed to the natural course of the disease. However, executive function and attention improved only following HBOT. A previous study has demonstrated decreases in CBF in frontal and temporal cortices of post-COVID-19 patients^24^. Hence, the improvement following HBOT may be attributed to the increases in CBF and MD, demonstrated in the BA10, BA8 and BA6 areas that are associated with executive function and attention^25–27^.

Post-COVID-19 condition is associated with long term psychiatric symptoms including depression, anxiety, and somatization^3,4^. HBOT improved both depression and somatization symptoms. Benedetti et al. detected robust associations between anxiety and depression in post-COVID-19 patients, and DTI measures of GM and WM microstructure in the superior and posterior corona radiata, superior longitudinal fasciculus and cingulum^28^. In this study, the psychiatric improvement was also associated microstructure changes in the superior corona radiata area. Furthermore, we previously studied childhood abuse induced fibromyalgia patients in whom HBOT induced significant metabolic improvements in the same brain areas in addition to similar clinical improvement in somatization and depression^14^. The association between improvements in the psychiatric symptoms to the MRI changes gives further strength to the biological nature of this disease and HBOT’s effect.

HBOT also improved pain interference. Interestingly, the pain interference score was high at baseline in both groups whereas the severity score was not. Diffuse muscle and joint pain without local inflammation or malformation is one of the common symptoms of post-COVID-19, resembling other central sensitization syndromes, such as fibromyalgia. A growing number of clinical studies, have demonstrated the efficacy of HBOT in improving pain and quality of life of fibromyalgia patients^14,15,29–32^. Previous studies have shown that fibromyalgia is associated with decreased brain perfusion in the insula, hippocampus, putamen, prefrontal and cingulate cortex^33–35^. In the current study, these regions showed increased perfusion after HBOT.

In post-COVID-19 condition, fatigue is a common symptom, and this symptom was reported in 77% of the study’s patients. HBOT improved both physical limitations and the energy domains. In concordance, Robbins et al. reported a significant improvement in fatigue following HBOT sessions in post-COVID-19 patients^22^. The HBOT induced MD changes in the frontal lobe (BA 6,8,10) can be associated with the clinical results, as hypometabolism in the frontal lobe has been implicated with fatigue in COVID-19 patients^36^. Post-COVID-19 fatigue has many overlaps with chronic fatigue syndrome (CFS). Symptoms common to CFS and post-COVID-19 condition include fatigue, pain, neurocognitive/psychiatric symptoms, reduced daily activity, and post-exertional malaise^36^. Previous studies have demonstrated the efficacy of HBOT in CFS, in reducing symptom severity and increasing quality of life^37,38^.

The pathogenesis of post-COVID-19 condition in the central nervous system includes direct neuronal injury in the frontal lobes, chronic injury mediated by glial cells, ischemic events mediated by thrombotic events, mitochondrial dysfunction, and chronic inflammation^11–19^. Growing evidence shows that new HBOT protocols can induce neuroplasticity and improve brain function even months to years after the acute injury^12,14–18^. These protocols, including the one used in the current study, utilize the so called “hyperoxic-hypoxic paradox”, by which repeated fluctuation in both pressure and oxygen concentrations induce gene expression and metabolic pathways that are essential for regeneration without the hazardous hypoxia^11^. These pathways can modulate the immune system, promote angiogenesis, restore mitochondrial function and induce neurogenesis in injured brain tissue^11–19^. Some or all of these effects may explain the beneficial effects found in the current study.

The primary strength of this study is the sham protocol which was found effective in blinding participants to treatment. Although this study presents advanced imaging methods, and whole brain study approach, which were correlated with clinical findings, the study has several limitations. The sample size is relatively small. Larger cohort studies may identify patients who can benefit the most from the treatment. The HBOT protocol included 40 sessions. However, an optimal number of sessions for maximal therapeutic effect has yet to be determined. Lastly, results were collected 1–3 weeks after the last HBOT session, and long-term results remain to be collected.

In conclusion, HBOT can improve dysexecutive functions, psychiatric symptoms (depression, anxiety and somatization), pain interference symptoms and fatigue of patients suffering from post-COVID-19 condition. The beneficial effect can be attributed to increased brain perfusion and neuroplasticity in regions associated with cognitive and emotional roles. Further studies are needed to optimize patient selection and to evaluate long-term outcomes.

## Methods

### Patients

Patients were ≥ 18 years old with reported post-COVID-19 cognitive symptoms that affected their quality of life and persisted for more than three months following an RT-PCR test confirming a symptomatic SARS-CoV-2 infection. Patients were excluded if they had a history of pathological cognitive decline, traumatic brain injury or any other known non-COVID-19 brain pathology. The inclusion and exclusion criteria are listed Supplementary information.

### Trial design

A prospective randomized, double blind, sham-controlled, phase II exploratory study was conducted from December 14, 2020, to December 27, 2021, at Shamir Medical Center (SMC), Israel. After signing an informed consent, patients were randomized to either HBOT or sham-control groups in a 1:1 ratio according to a computerized randomization table, supervised by a blinded researcher. To evaluate participant masking, patients were questioned after the first session on their perception regarding the treatment they received. Evaluation procedure was done at baseline and 1–3 weeks after the last HBOT/control session. All evaluators were blinded to the patients’ group allocation. The study was approved by SMC’s Institutional Review Board (IRB) (No. 332-20-ASF) and all participants signed an informed consent prior to their inclusion. All research was performed according to the relevant guidelines and regulations. This study was registered with ClinicalTrials.gov, number NCT04647656 on 01/12/2020.

### Intervention

Both HBOT and sham protocols were administrated in a multi-place Starmed-2700 chamber (HAUX, Germany). The protocol comprised of 40 daily sessions, five sessions per week within a two-month period. The HBOT protocol included breathing 100% oxygen by mask at 2ATA for 90 min with five-minute air breaks every 20 min. Compression/decompression rates were 1.0 m/min. The sham protocol included breathing 21% oxygen by mask at 1.03 ATA for 90 min. To mask the controls, the chamber pressure was raised up to 1.2 ATA during the first five minutes of the session along with circulating air noise followed by decompression (0.4 m/min) to 1.03 ATA during the next five minutes.

### Primary and secondary outcomes

The primary outcome of the study was the cognitive assessment as evaluated by the Mindstreams computerized cognitive testing battery (NeuroTrax Corporation, Bellaire, TX). This assessment evaluates various cognitive domains including: memory, executive function, attention, information processing speed, and motor skills. Cognitive scores were normalized for age, gender and educational levels. The tests methods are described in the Supplementary information.

The secondary outcomes include the following measures:

Brain imaging MRI scans were performed on a MAGNETOM VIDA 3 T scanner, configured with 64-channel receiver head coils (Siemens Healthcare, Erlangen, Germany). The MRI protocol included T2-weighted, 3D fluid attenuated inversion recovery (FLAIR), susceptibility weighted imaging (SWI), pre- and post-contrast high-resolution MPRAGE 3D T1-weighted, dynamic susceptibility conSupplementary informationtrast (DSC) for calculating whole-brain quantitative perfusion maps, and diffusion tensor imaging (DTI) for microstructure changes in grey and white matter determination. A detailed description is found in the . Briefly, preprocessing of DSC and DTI images was performed using the SPM software (version 12, UCL, London, UK) and included motion correction, co-registration with MPRAGE T1 images, spatial normalization, and spatial smoothing with a kernel size of 6 mm full width half maximum (FWHM). Whole-brain quantitative perfusion analysis was performed as described in previous studies^39,40^. MR signal intensity was converted to Gd concentrations, AIF was determined automatically, fitted to the gamma variate function and deconvolved on a voxel-by-voxel basis to calculate brain perfusion maps.

Diffusion brain volumes denoising was performed using Joint Anisotropic LMMSE Filter for Stationary Rician noise removal^41^ and calculation of DTI-FA (fractional anisotropy) and MD (mean diffusivity) maps were performed using an in-house software written in Matlab R2021b (Mathworks, Natick, MA).

Included self-reported questionnaires were the short form-36 (SF-36) to assess quality of life, the Pittsburgh Sleep Quality Index (PSQI) to assess sleep quality, the Brief Symptom Inventory (BSI-18) to evaluate psychological distress, based on three subscales: depression, anxiety, and somatization, and the Brief Pain Inventory (BPI) to measure pain intensity and impact.

The sense of smell was evaluated by the Sniffin’ Sticks Test (Burghardt, Wedel, Germany). The kit is standardized for age and gender. Taste was evaluated by a Taste Strip Test (Burghardt, Wedel, Germany), including four tastes: bitter, sour, salt and sweet.

Pulmonary function measurements were performed by a KoKo Sx1000 spirometer (Nspire health, USA). Blood samples were collected for complete blood count, chemistry and inflammatory markers. Participants were monitored for adverse events including barotraumas (either ear or sinuses), and oxygen toxicity (pulmonary and central nervous system). This article discusses cognitive and behavioral aspects of post-COVID-19 condition. Additional secondary outcomes including neuro-physical evaluation, cardiopulmonary exercise test, echocardiography, and functional brain imaging will be presented in future manuscripts.

### Statistical analysis

Continuous data are expressed as means ± standard deviations (SD). Two-tailed independent t-tests with were performed to compare variables between groups when a normality assumption held according to a Kolmogorov–Smirnov test. Net effect sizes were evaluated using Cohen's d method, defined as the improvement from baseline after sham intervention was subtracted from the improvement after HBOT, divided by the pooled standard deviation of the composite score. Categorical data were expressed in numbers and percentages, compared by chi-square/Fisher’s exact tests. To evaluate HBOT’s effect, a mixed-model repeated-measure ANOVA model was used to compare post-treatment and pre-treatment data. The model included time, group and the group-by-time interaction. A Bonferroni correction was used for the multiple comparisons. A value of p < 0.05 was considered significant. Pearson’s correlations were performed between perfusion and diffusion changes and the change in questionnaire scores before and after HBOT and sham. Imaging data analysis was performed on the normalized CBF, FA and MD maps, using the voxel-based method to generate statistical parametric maps. A gray matter mask was applied on the CBF and MD maps, and a white matter mask on the FA maps (using a threshold of 0.2). A within-subject repeated measure ANOVA model was used to test the main interaction effect between time and group implemented in SPM software (version 12, UCL, London, UK). A sequential Hochberg correction was used to correct for multiple comparisons (P < 0.05)^42^. Data analysis was performed using Matlab R2021b (Mathworks, Natick, MA) Statistics Toolbox.

The estimated sample size was calculated based on our recent study in healthy adults^19^. A Mindstreams-NeuroTrax global cognitive score improvement of 5.2 and 0.8 points, with a standard deviation of 6.7 points was found in the HBOT and control groups respectively. Assuming a power of 80%, and 5% two-sided level of significance, a total of 74 participants would be required, 37 participants in each arm. Considering a dropout rate of 15% the total sample size required is 85.

## Supplementary Information

Supplementary Information.

## Data Availability

The datasets analyzed during the current study available from the corresponding author on reasonable request.
