# Supplementary material for: Hyperbaric oxygen therapy improves neurocognitive functions and symptoms of post-COVID condition: randomized controlled trial
Source: Sci Rep. 2022 Jul 12;12:11252. doi: 10.1038/s41598-022-15565-0 (PMC9276805; doi:10.1038/s41598-022-15565-0)
Supplement: Supplementary file 1 — Supplementary Information. [file 41598_2022_15565_MOESM1_ESM.pdf]

Hyperbaric oxygen therapy improves neurocognitive functions and symptoms of post-COVID condition: randomized controlled trial.

Shani Zilberman-Itskovich[1,2†], Merav Catalogna[1†], Efrat Sasson[1], Karin Elman-Shina[1,2], Amir Hadanny[1,2], Erez Lang[1,2], Shachar Finci[1,2], Nir Polak[1,2], Gregory Fishlev[1,2], Calanit Korin[1,2], Ran Shorer[1], Yoav Parag[1], Marina Sova[1], and Shai Efrati[1,2,3]

SUPPLEMENTARY INFORMATION

Table of contents

1 Methods 2
1.1 Inclusion criteria 2
1.2 Exclusion criteria 2
1.3 The NeuroTrax cognitive battery test 3
1.4 MRI protocol and analysis 5
1.5 References 8
2 Supplementary Tables 9
Table 1: Baseline symptoms 9
Table 2: Covid-19 infection symptoms 10
Table 3: Neurocognitive performance changes ANOVA (group x time) 10
Table 4: Questionnaire scores analysis - ANOVA (group x time) 11
Table 5: Brain regions with significant perfusion (CBF) increases in gray matter 12
Table 6: Brain regions with significant DTI-MD increases in gray-matter 13
Table 7: Brain regions with significant DTI-FA increases in white-matter 13
Table 8: Smell and taste changes 14
Table 9: Spirometry test changes 15
Table 10: Chemistry blood tests 16
Table 11: Adverse events during treatment period 17
3 Supplementary Figures 18
Figure 1. Study flowchart and timeline 18
Figure 2. SHAM test results matrix 19
Figure 3. Smell total score changes in HBOT and control arms 20
Figure 4. Taste total score changes in HBOT and control arms 21

1 Methods

1.1 Inclusion criteria

- Age above 18 years
- Reported post-COVID-19 cognitive deterioration that affects the quality of life and has persisted for at least three months after a confirmed infection. Confirmed infection was established by a positive PCR test for COVID-19 along with clinical signs and symptoms of acute infection (at least two of the following: fever, cough, sputum, sore throat, muscle pain, diarrhea, leukopenia (under 500 cells) or low oxygen saturation).
- Subject is willing and able to read, understand and sign an informed consent.

1.2 Exclusion criteria

- Inability to attend scheduled clinic visits and/or comply with the study protocol
- History of TBI or any other non-COVID brain pathology
- Active malignancy
- Substance use at baseline
- Severe or unstable physical disorders or major cognitive deficits at baseline
- HBOT for any reason prior to study enrolment
- Chest pathology incompatible with pressure changes (including moderate to severe asthma)
- Ear or sinus pathology incompatible with pressure changes
- An inability to perform an awake brain MRI

1.3 The NeuroTrax cognitive battery test

The primary endpoint of the study was a cognitive health assessment as evaluated by the NeuroTrax computerized cognitive testing battery (NeuroTrax Corporation, Bellaire, TX) [1,2]. This assessment comprises of several cognitive tests that evaluate various aspects of brain capabilities including: memory, executive function, attention, information processing speed, and motor skills. In the current study, the cognitive index was based on scores of six cognitive tests:

1. Verbal memory: Ten pairs of words are presented, followed by a recognition test in which the first word of a previously presented pair appears together with a list of four words from which the participant chooses the other member of the pair. There are four immediate repetitions and one delayed repetition after 10 minutes.
2. Non-verbal memory. Eight pictures of simple geometric objects are presented, followed by a recognition test in which four versions of each object are presented, each oriented in a different direction. There are four immediate repetitions and one delayed repetition after 10 minutes.
3. Go–no-go test. In this continuous performance test, a colored square (red, green, white or blue) appears randomly on the center of the screen. The participant is asked to respond quickly, only to red squares, by pressing the mouse button, and not to react to the presentation of any other colored square.
4. Stroop test. Timed test of response inhibition, modified from the Stroop paper-based test. In the first phase, participants choose a colored square matching the color of a general word (for example, the word "Cat" appears in red letters; the participant must choose the red square of two-colored squares in the following screen). In the next phase (termed the choice reaction time test), the task is to choose the colored square matching the name of the color presented in white letter–color. In the final (Stroop interference) phase, participants are asked to choose the colored square that matches the color and not the meaning of a former color-naming word, presented in an incongruent color (for example, the word "RED" appears in green letters, the patient is asked to choose the color green and not red, a task requiring the ability to inhibit an automatic response to the meaning of the word).
5. Staged information processing test. A timed test requiring a reaction based on solving simple arithmetic problems (pressing the right/left mouse button if the answer is higher/lower than 4, respectively), with three levels of information processing load (single-digit, two-digit addition/subtraction and three-digit addition/subtraction problems), each containing three speed levels (3, 2, and 1 second for the presentation of the stimuli).

6. Catch game. A test of motor planning that requires participants to “catch” a falling object on a computer screen by moving a paddle horizontally.

The cognitive domains are combined of several sub-tests, as detailed in the table below. The global score is the mean value of the cognitive domains.

Cognitive Domains and Sub-Test Measures

Domain	Description
Memory	Verbal memory: Total accuracy<br>Delayed verbal memory: Accuracy<br>Non-verbal memory: Total accuracy<br>Delayed non-verbal memory: Accuracy
Executive function	Go-no-go: Composite score<br>Stroop: Composite score, level 3<br>Catch Game: Total score
Attention	Go-no-go: Response time<br>Go-no-go: Response time standard deviation<br>Stroop interference: Response time, level 2<br>Staged information processing speed: Response time, level 1.2<br>Staged information processing speed: Accuracy, level 2.3
Information processing speed	Staged information processing speed: Composite scores, levels 1.1, 1.3, 2.1 and 2.2
Motor skills	Finger tapping: Inter-tap interval<br>Finger tapping: Tap interval standard deviation<br>Catch game: Time to make first move

The assigned scores are uploaded to the NeuroTrax central server. Outcome parameters are calculated using custom software blind to diagnosis or the testing site. To minimize differences related to age and education, each outcome parameter is normalized and fit to an IQ-like scale (mean=100, STD=15), according to the participants’ age and education. We note that the scores are evaluated according to normative data from cognitively healthy individuals, collected in controlled research studies that were conducted at more than 10 clinical sites [2]. Additional information is also available on the NeuroTrax website (http://www.neurotrax.com/).

1.4 MRI protocol and analysis

MRI scans were performed on a MAGNETOM VIDA 3T scanner, configured with 64-channel receiver head coils (Siemens Healthcare, Erlangen, Germany). The MRI protocol included T2-weighted, 3D fluid attenuated inversion recovery (FLAIR), susceptibility weighted imaging (SWI), pre- and post-contrast high-resolution MPRAGE 3D T1-weighted, dynamic susceptibility contrast (DSC), and diffusion tensor imaging (DTI).

MRI scans sequences parameters

DSC-MRI: Fifty T2*-weighted gradient-echo echo planar imaging (EPI) volumes were acquired, two repetitions before a bolus injection of gadolinium-DTPA (Gd-DTPA, 0.2 ml/kg, administered at 5 ml/sec), 48 repetitions after injection of Gd-DTPA. Sequence parameters: TR: 2,500 ms, TE: 30 ms, flip angle: 30°, voxel size: 1.8 x1.8, Matrix: 128x128, number of slices: 35, and slice thickness = 3 mm.

DTI: Whole brain diffusion weighted images were acquired with the following parameters: Sixty axial slices, slice thickness = 2 mm, voxel size = 2 x 2 mm, TR = 3400 ms, TE = 63 ms, and matrix = 248 x 128 mm, SMS factor = 3. Diffusion gradients were applied along 64 noncollinear directions (b = 1000 s/mm2) and seven volumes without diffusion weighting, including five volumes in read directions and two volumes in phase direction to compensate for EPI distortions.

MPRAGE was acquired in sagittal orientation with 1 mm isotropic resolution. Sequence parameters: TR: 2,000 ms, TE: 1.9 ms, flip angle: 9°, TI: 920 ms, FOV: 256 x 256, and 256 contiguous slices.

DSC-MRI analysis

Whole-brain quantitative perfusion analysis was performed as described in previous studies [3,4]. MR signal intensity was converted to Gd concentrations, AIF was determined automatically, fitted to the gamma variate function and deconvolved on a voxel-by-voxel basis to calculate the CBF, CBV, and MTT maps according to the following steps:

1. Conversion of signal intensity to concentration of Gd-DTPA with respect to time:

$$C_m(t) = -K * \ln(\frac{S(t)}{S_0})$$

where $C_m(t)$ is the measured concentration of Gd-DTPA with respect to time, K is a proportionality constant that is inversely proportional to the TE and depends on the MR scanner, S(t) is the MRI signal intensity with respect to time, and S_o is the baseline MRI

signal before the presence of Gd-DTPA and after a steady-state magnetization has been achieved [5].

2. Arterial input function (AIF): the AIF was measured automatically, using the following algorithm:
    a. The volume with maximum $C_m(t)$ intensity was identified (10th-13th volume). Only voxels with maximum intensity in this volume were identified as AIF candidates.
    b. Only voxels with maximum intensity higher than the 96th percentile and lower than the 99.9^{th} percentile were included.
    c. Only voxels with a shape of sharp increase and sharp decrease were included.
    d. The AIF voxel candidates were fitted to the gamma variate function using the following equation[5]. Goodness of fit was evaluated and only voxels with $R^2 > 0.96$ were included.

    $$AIF_{fit}(t) \ or \ C_{fit}(t) = -K(x-\Delta)^{\alpha} * e^{-\frac{x-\Delta}{B}} * F_{step}(x-\Delta)$$

    e. The final AIF was an average of the $C_m(t)$ signal in the voxels passing the above criteria.
    f. Normalization of AIF: To allow a uniform time of injection in all subjects and DSC scans, the $C_m(t)$ was shifted in case of early/late injection to allow a uniform AIF peak at the 10th volume.
3. Gamma fitting of AIF and Cm: The AIF and $C_m(t)$ were fitted to the gamma variate function using the gamma fit equation (see above) [5], where $AIF_{fit}(t)$ and $C_{fit}(t)$ are the fitted AIF(t) and $C_m(t)$ curves, respectively, K is a constant, x is the image number, Δ is the delay between image 0 and the arrival of the bolus (a

positive number), a and B are gamma variate parameters, and F_{step} is a step function defined by:

$$F_{step} = \begin{cases} 1 & for\ (x - \Delta) \geq 0 \\ 0 & for\ (x - \Delta) < 0 \end{cases}$$

4. SVD deconvolution: The fitted AIF was used to calculate C(t) (the tissue response to an instantaneous arterial bolus) using SVD deconvolutionas[6]. In short, the values for the AIF and $C_m(t)$ curves can be written in vector notation as C = AIF−1 • Cm, where C represents the matrix of the deconvolved C(t) curve. This equation can be solved using the SVD technique, whereby the matrix AIF is decomposed into three matrices AIF = U • W • VT. The inverse of AIF can be calculated as AIF^{-1} = V • [diag(1/wj)] • UT, where [diag(1/wj)] represents the reciprocals of the diagonal elements of W. When calculating AIF^{-1}, problems arise when W contains singular values (i.e., wj = 0 or is close to 0) and will cause the curve C(t) to oscillate. Therefore, we used a cutoff threshold of 10% [7].
5. Calculation of CBV was performed based on the fitted $C_m(t)$ and AIF:

$$CBV = \frac{k}{\rho} * \frac{\int C_m(t)\,dt}{\int AIF(t)\,dt}$$

where κ = (1 − HCTLV)/(1 − HCTSV) corrects for the fact that the hematocrit in large vessels (HCTLV was set to 0.45) is larger than the hematocrit of small vessels (HCTSV was set to 0.25) and ρ is the density of brain tissue (1.04 g/ml) [5].

6. Calculation of CBF was performed using the following equation:

$$\frac{CBV}{CBF} = \frac{\int C(t)\,dt}{C_{max}}$$

where C(t) is the concentration of Gd-DTPA in a tissue region and C_{max} is the maximum of this curve [5].

7. MTT was calculated as:

$$MTT = \frac{CBV}{CBF}$$

8. Normalization of the CBF: Since the amount of injection was not uniform between scans, the CBF was normalized using a factor of 1.6 divided by the AIF peak value.

Perfusion maps were performed using an in-house software written in Matlab R2021b (Mathworks, Natick, MA).

1.5 References

1 Doniger, G. M. *Mindstreams Computerized Cognitive Tests: Test Descriptions. Available: http://www.mirror.upsite.co.il/uploaded/files/1383_e7d7d3d98c924f036d3123733419149d.pdf. Accessed 05 July 2013*, <http://www.mirror.upsite.co.il/uploaded/files/1383_e7d7d3d98c924f036d3123733419149d.pdf> (2007).

2 Doniger, G. M. *Guide to MindStreams Normative Data. Available: http://www.mirror.upsite.co.il/uploaded/files/1383_b44d4786c91058be301cb09a94ba70f4.pdf. Accessed 05 July 2013*, <http://www.mirror.upsite.co.il/uploaded/files/1383_b44d4786c91058be301cb09a94ba70f4.pdf> (2012).

3 Østergaard, L. *et al.* High resolution measurement of cerebral blood flow using intravascular tracer bolus passages. Part II: Experimental comparison and preliminary results. *Magnetic resonance in medicine* **36**, 726-736 (1996).

4 Ostergaard, L., Weisskoff, R. M., Chesler, D. A., Gyldensted, C. & Rosen, B. R. High resolution measurement of cerebral blood flow using intravascular tracer bolus passages. Part I: Mathematical approach and statistical analysis. *Magn Reson Med* **36**, 715-725, doi:10.1002/mrm.1910360510 (1996).

5 Smith, A. M., Grandin, C. B., Duprez, T., Mataigne, F. & Cosnard, G. Whole brain quantitative CBF, CBV, and MTT measurements using MRI bolus tracking: implementation and application to data acquired from hyperacute stroke patients. *J Magn Reson Imaging* **12**, 400-410 (2000).

6 Østergaard, L., Weisskoff, R. M., Chesler, D. A., Gyldensted, C. & Rosen, B. R. High resolution measurement of cerebral blood flow using intravascular tracer bolus passages. Part I: Mathematical approach and statistical analysis. *Magnetic resonance in medicine* **36**, 715-725 (1996).

7 Ostergaard, L. *et al.* High resolution measurement of cerebral blood flow using intravascular tracer bolus passages. Part II: Experimental comparison and preliminary results. *Magn Reson Med* **36**, 726-736 (1996).

2 Supplementary Tables

Table 1: Baseline symptoms*

Symptom	HBOT	Control	P-value
N	37	36	
Fatigue	26 (70.3)	30 (83.3)	0.269
Concentrating	28 (75.7)	25 (69.4)	0.607
Sleep	27 (73.0)	24 (66.7)	0.616
Forgetfulness	24 (64.9)	26 (72.2)	0.616
Finding words	19 (51.4)	23 (63.9)	0.346
Quality of sleep	27 (73.0)	28 (77.8)	0.787
Muscle aches	20 (54.1)	21 (58.3)	0.815
Joint aches	15 (40.5)	19 (52.8)	0.352
Anxiety	7 (18.9)	10 (27.8)	0.417
Sadness	6 (16.2)	12 (33.3)	0.109
Swallowing	3 (8.1)	3 (8.3)	1.000
Taste	9 (24.3)	6 (16.7)	0.564
Smell	9 (24.3)	8 (22.2)	1.000
Loss of appetite	7 (18.9)	3 (8.3)	0.308
Everyday activities	13 (35.1)	14 (38.9)	0.811
Strained activities	22 (59.5)	26 (72.2)	0.326
Confusion	11 (29.7)	10 (27.8)	1.000

* Self-reported

Table 2: Covid-19 infection symptoms

Symptom	HBOT	Control	P-value
N	37	36	
Abdominal pain	1 (2.7)	0 (0.0)	1.000
Chills	1 (2.7)	0 (0.0)	1.000
Dry cough	10 (27.0)	16 (44.4)	0.147
Diarrhea	8 (21.6)	3 (8.3)	0.190
Dyspnea	10 (27.0)	12 (33.3)	0.616
Fever ≥ 38˚c	22 (59.5)	24 (66.7)	0.630
Headache	12 (32.4)	11 (30.6)	1.000
Joint ache	1 (2.7)	0 (0.0)	1.000
Problem in taste sensation	16 (43.2)	7 (19.4)	0.043
Problem in smell sensation	12 (32.4)	5 (13.9)	0.096
Low saturation	4 (10.8)	7 (19.4)	0.345
Muscle aches	23 (62.2)	21 (58.3)	0.813
Myalgia	1 (2.7)	1 (2.8)	1.000
Sore throat	4 (10.8)	5 (13.9)	0.736
Sputum	0 (0.0)	2 (5.6)	0.240
Fatigue	4 (10.8)	3 (8.3)	1.000

Table 3: Neurocognitive performance changes ANOVA (group x time)

	**Main effect of group**		**Main effect of time**		**Interaction effect**	
	F	P-value	F	P-value	F	P-value
Score	0.33	0.566	26.88	0.000	4.47	0.038
Memory	0.07	0.793	43.79	0.000	0.23	0.636
Executive function	1.82	0.182	10.85	0.002	4.16	0.045
Attention	0.00	0.978	3.12	0.082	3.91	0.052
Information processing speed	0.47	0.495	16.52	0.000	1.67	0.200
Motor skills	0.22	0.637	2.23	0.140	2.08	0.154

Table 4: Questionnaire scores analysis - ANOVA (group x time)

	Main effect of group		Main effect of time		Interaction effect	
	**F**	**P-value**	**F**	**P-value**	**F**	**P-value**
**SF-36**						
Physical functioning	1.422	0.237	5.482	0.022	1.322	0.254
Physical limitations	0.001	0.970	17.754	0.000	5.430	0.023
Emotional limitations	0.570	0.453	18.927	0.000	0.846	0.361
Energy	1.707	0.196	19.378	0.000	4.976	0.029
Emotional wellbeing	0.760	0.386	12.819	0.001	3.841	0.054
Social function	0.047	0.830	25.863	0.000	2.795	0.099
Pain domain	0.074	0.787	25.913	0.000	1.179	0.281
General health domain	5.241	0.025	7.472	0.008	2.088	0.153
**PSQI**						
Global	0.190	0.664	23.453	0.000	4.302	0.042
Sleep quality	0.625	0.432	19.472	0.000	1.753	0.190
Sleep latency	0.462	0.499	25.595	0.000	1.730	0.193
Sleep duration	0.006	0.939	0.282	0.597	2.364	0.129
Sleep efficiency	0.021	0.884	3.767	0.056	0.041	0.840
Sleep disturbances	0.038	0.845	12.138	0.001	3.940	0.051
Sleep medication	0.019	0.892	1.737	0.192	1.150	0.287
Daytime dysfunction	0.232	0.631	13.821	0.000	0.891	0.348
**BSI-18**						
Total	0.056	0.813	16.799	0.000	7.372	0.008
Somatization	0.037	0.849	12.457	0.001	6.312	0.014
Depression	0.029	0.866	11.792	0.001	4.395	0.040
Anxiety	0.065	0.800	7.036	0.010	3.169	0.079
**BPI**						
Pain severity score	0.117	0.734	0.465	0.498	0.011	0.917
Pain interference score	0.021	0.884	13.103	0.001	11.204	0.001

Table 5: Brain regions with significant perfusion (CBF) increases in gray matter

		MNI Coordinates					
**Anatomical location**	**BA**	**X**	**Y**	**Z**	**t-value**	**Cluster size**	**P value**
Supramarginal Gyrus R (Parietal)	40	61	-36	47	4.47	306	0.000008*
Superior Parietal Lobule R (Parietal)	7	42	-62	52	4.46	138	0.000008*
Parahippocampal Gyrus L		-24	-14	-9	4.29	396	0.000009*
Insula R	13	42	17	0	4.22	247	0.000012*
Supplementary Motor Area L (Frontal)	6	-6	20	66	4.21	197	0.000013*
Supramarginal Gyrus L (Parietal)	40	-46	-53	54	4.33	193	0.000015*
Supramarginal Gyrus L (Parietal)	40	-63	-22	27	4.32	177	0.000015*
Anterior Cingulate Gyrus\ Medial Superior Frontal Gyrus L	10/32	-4	50	9	4.32	188	0.000026*
Anterior Cingulate Gyrus\ Dorsal Prefrontal R	32 \9	4	35	24	3.96	413	0.000037
Putamen R		29	-6	-3	3.95	229	0.000039
Frontal Operculum \ Insula R	13	35	-24	21	3.93	287	0.000042
Inferior Frontal Gyrus \ Lateral orbitofrontal							
Cortex L	47	-40	31	-13	3.92	89	0.000044
Temporal pole \ Middle Temporal Gyrus L	38	-38	11	37	3.86	219	0.000056
Postcentral Gyrus L (Parietal)	3	-46	-17	40	3.76	162	0.000086
Superior Temporal Gyrus L	42	-62	-29	6	3.73	81	0.000097
Hippocampus L		-17	-8	-11	3.75	52	0.000130
Insula L	13	-44	6	6	3.73	53	0.000137
Fusiform Gyrus R	37	-62	-52	2	3.72	73	0.000143
Anterior Insula L	13	-40	16	-8	3.7	157	0.000153
Inferior Frontal Gyrus \ Posterior Orbitofrontal Cortex R	47	26	26	-20	3.63	68	0.000197
Putamen L		-20	4	-6	3.62	69	0.000207
Inferior Frontal Gyrus L	45	-44	18	3	3.58	57	0.000239

The table reports each brain region which was found significant in a time-by-group repeated measures ANOVA comparing the two groups. The results are shown in specific Montreal Neurological Institute (MNI) coordinates; X, sagittal, Y, coronal, Z, axial, refers to Montreal Neurological Institute. BA, Brodmann area. *significant after correction to multiple comparisons, $p<0.05$; All coordinates emerged at a threshold of $P < 0.0005$, uncorrected. R, right; L, left; CBF, cerebral blood flow.

Table 6: Brain regions with significant DTI-MD increases in gray-matter

		MNI Coordinates					
**Anatomical location**	**BA**	**X**	**Y**	**Z**	**t-value**	**Cluster size**	**P value**
Frontal Precentral Gyrus L	6	-33	-8	62	4.58	454	0.000005*
Middle Frontal Gyrus R	10	38	50	28	4.35	233	0.000013*
Middle Frontal Gyrus R	8	38	16	57	3.99	325	0.000052*
Superior Frontal Gyrus L	10	-12	65	3	3.94	119	0.000064
Superior Frontal Gyrus L	6	-16	2	72	3.9	366	0.000075
Medial Superior Frontal Gyrus R	8	7	32	48	3.61	282	0.000215
Inferior Parietal Lobule L	40	51	-40	57	3.47	33	0.000350
Middle Frontal Gyrus \Middle Orbital Gyrus L	10	9	65	-1	3.34	76	0.000536
Post Central Gyrus R (S1)	1	67	-11	14	3.19	26	0.000863

The table reports each brain region which was found significant in a time-by-group repeated measures ANOVA comparing the two groups. The results are shown in specific Montreal Neurological Institute (MNI) coordinates; X, sagittal, Y, coronal, Z, axial, refers to Montreal Neurological Institute. BA, Brodmann area. *significant after correction to multiple comparisons, $p<0.05$; All coordinates emerged at a threshold of $P < 0.002$, uncorrected. R, right; L, left; MD, mean diffusivity

Table 7: Brain regions with significant DTI-FA increases in white-matter

	MNI Coordinates					
**Anatomical location**	**X**	**Y**	**Z**	**t-value**	**Cluster size**	**P value**
Superior Corona Radiata R (Frontal)	20	-8	51	4.34	602*	0.00006*
Superior Corona Radiata L (Frontal)	-26	-16	51	3.96	345*	0.00006*
Superior Longitudinal Fasciculus L (Parietal)	-29	-53	35	3.83	74	0.00010
U fibers to SMA\ WM Superior Corona Radiata L (Frontal)	-16	1	54	3.78	233	0.00014
Pontine Crossing Tract- BS R	6	-28	-37	3.73	173	0.00014
Cingulum R	19	5	48	3.51	79	0.00030
Sagittal Stratum L (Temporal)	-44	-25	3	3.43	110	0.00039
External Capsule L (near Insula L)	-32	-17	6	3.29	36	0.00070
External Capsule R (near Putamen R)	34	-13	2	3.2	74	0.00070
Superior Corona Radiata R (Frontal)	-21	-12	23	3.26	187	0.00075
External Capsule R (near Insula R)	35	12	-6	3.24	81	0.00075

The table reports each brain region which was found significant in a time-by-group repeated measures ANOVA comparing the two groups. The results are shown in specific Montreal Neurological Institute (MNI) coordinates; X, sagittal, Y, coronal, Z, axial, refers to Montreal Neurological Institute. BA, Brodmann area. *significant after correction to multiple comparisons, $p<0.05$; All coordinates emerged at a threshold of $P < 0.002$, uncorrected. R, right; L, left; FA, fractional anisotropy

Table 8: Smell and taste changes

	HBOT					Control					ANOVA (group-by-time) Interaction			
	**N**	**Pre**	**Post**	**Change**	**Two months P-value**	**N**	**Pre**	**Post**	**Change**	**Two months P-value**	**P-value Baseline**	**Net effect size***	**F**	**P-value**
Smell														
All	37	9.1±1.9	10.1±2.1	0.9±2.0	0.006	36	9.3±2.3	9.6±2.2	0.4±1.6	0.156	0.780	0.311	1.76	0.189
Abnormal at baseline	27	8.3±1.6	9.6±2.2	1.3±2.2	0.005	25	8.3±2.2	9.2±2.4	0.9±1.7	0.0143	0.9653	0.215	0.597	0.443
Taste														
All	37	8.3±3.2	9.1±2.9	0.8±2.4	0.068	36	9.2±2.5	9.0±2.5	-0.2±2.3	0.6679	0.212	0.388	2.749	0.102
Sweet	37	2.0±1.0	2.7±1.3	0.7±1.2	0.001	37	2.2±1.0	2.6±1.1	0.4±1.0	0.0199	0.346	0.259	1.226	0.272
Sour	37	1.9±0.7	2.0±0.8	0.1±1.0	0.737	37	2.1±0.7	1.8±0.8	-0.3±1.1	0.1426	0.429	0.318	1.850	0.178
Salty	37	2.0±1.1	1.8±0.9	-0.1±1.3	0.523	37	2.1±1.1	1.8±1.1	-0.3±1.2	0.177	0.611	0.115	0.241	0.625
Bitter	37	2.4±1.4	2.5±1.3	0.1±1.0	0.431	37	2.8±1.1	2.8±1.2	-0.0±1.3	0.8968	0.216	0.141	0.361	0.550
Abnormal at baseline														
Total	18	5.6±2.2	7.6±2.9	2.0±2.4	0.003	12	6.3±1.5	6.8±1.8	0.4±2.7	0.6007	0.347	0.626	2.825	0.104
Sweet	18	1.3±0.7	2.2±1.4	0.9±1.2	0.007	12	1.4±0.6	2.3±1.1	0.8±1.2	0.0341	0.744	0.046	0.015	0.903
Sour	18	1.6±0.7	1.8±0.8	0.2±1.1	0.528	12	1.8±0.7	1.4±0.9	-0.4±1.4	0.3177	0.405	0.480	1.656	0.209
Salty	18	1.3±0.7	1.6±1.0	0.3±1.2	0.350	12	1.3±0.9	1.3±0.9	0.0±1.0	1.0000	0.930	0.240	0.414	0.525
Bitter	18	1.4±1.1	2.1±1.4	0.7±1.0	0.014	12	1.8±0.9	1.8±1.1	0.0±1.1	1.0000	0.274	0.624	2.800	0.105

Data are presented as mean ± SD; Bold, significant after Bonferroni correction; * Cohen’s d net effect size

Table 9: Spirometry test changes

	HBOT				Control					ANOVA (group-by-time) Interaction		
	**Pre**	**Post**	**Two months P-value**	**Change**	**Pre**	**Post**	**Two months P-value**	**Change**	**P-value Baseline**	**Net effect size***	**F**	**P-value**
N	37				36							
IC	3.0±1.0	3.2±1.1	0.0788	0.2±0.6	2.9±0.8	3.2±0.7	0.1150	0.2±0.9	0.857	-0.074	0.094	0.760
%pred	107.4±21.3	114.1±23.0	0.1257	5.1±18.9	113.1±33.7	121.8±23.3	0.1450	8.7±34.9	0.408	-0.127	0.28	0.598
VC	3.6±1.1	3.7±1.1	0.1225	0.1±0.2	3.5±0.7	3.6±0.7	0.0225	0.1±0.3	0.586	-0.159	0.444	0.508
%pred	86.6±13.7	88.0±12.6	0.1258	1.4±5.3	88.5±13.1	91.0±12.4	0.0284	2.6±6.7	0.576	-0.183	0.586	0.447
IRV	2.0±1.0	2.3±0.9	0.1002	0.2±0.7	2.1±0.8	2.3±0.7	0.1972	0.2±0.9	0.585	-0.008	0.001	0.972
VT	1.0±0.6	1.0±0.6	0.7592	-0.0±0.6	0.8±0.4	0.8±0.5	0.7385	0.0±0.5	0.159	-0.110	0.208	0.650
FVC	3.5±1.1	3.6±1.0	0.5803	0.0±0.3	3.5±0.7	3.5±0.7	0.1923	0.0±0.2	0.701	-0.103	0.187	0.667
%pred	85.7±14.3	86.6±13.0	0.4272	0.8±6.1	88.6±12.3	90.0±13.0	0.1238	1.4±5.5	0.380	-0.106	0.2	0.656
FEV1	2.8±0.9	2.8±0.9	0.9657	-0.0±0.2	2.9±0.6	2.9±0.6	0.3062	0.0±0.2	0.802	-0.181	0.573	0.452
%pred	85.2±14.8	85.5±14.2	0.7763	0.3±6.5	91.9±13.5	93.3±13.8	0.1547	1.4±5.8	0.055	-0.181	0.573	0.452
FEV1/FVC	79.8±6.5	79.2±6.5	0.313	-0.6±3.5	83.1±5.3	82.6±5.0	0.6625	-0.3±4.0	0.025	-0.081	0.115	0.736
%pred	99.3±7.7	98.5±7.4	0.2858	-0.8±4.4	103.3±6.7	102.6±6.1	0.6946	-0.3±5.1	0.025	-0.096	0.162	0.689
PEF	6.8±2.2	6.9±2.1	0.5362	0.1±1.0	6.6±1.9	7.0±1.9	0.0433	0.4±1.3	0.641	-0.287	1.466	0.230
fef25-75	2.8±1.2	2.8±1.1	0.6918	-0.0±0.4	3.3±1.0	3.2±1.0	0.6588	-0.0±0.5	0.091	0.025	0.011	0.917
%pred	88.1±25.6	88.2±26.8	0.952	0.1±13.9	107.0±27.6	107.3±30.6	0.9105	0.3±16.2	0.004	-0.011	0.0020	0.964
fev1/vcmax	77.8±6.3	76.9±7.9	0.331	-0.9±5.7	81.5±6.0	79.1±8.7	0.1723	-2.1±8.9	0.016	0.155	0.422	0.518
%pred	96.8±7.3	95.6±9.3	0.3201	-1.2±7.2	101.2±7.1	99.8±6.2	0.3224	-1.0±5.9	0.014	-0.035	0.021	0.885

Data are presented as mean ± SD; Bold, significant after Bonferroni correction; * Cohen's d net effect size

Table 10: Chemistry blood tests

	HBOT				Control					ANOVA (group-by-time) Interaction		
	**Pre**	**Post**	**Two months P-value**	**Change**	**Pre**	**Post**	**Two months P-value**	**Change**	**P-value Baseline**	**Net effect size***	**F**	**P-value**
URAC-B	4.9±1.4	5.0±1.3	0.376	0.1±0.8	4.8±1.4	4.9±1.5	0.237	0.1±0.6	0.843	-0.011	0.002	0.964
OSMOLcal	289.4±3.6	288.5±5.0	0.404	-0.8±5.8	288.1±5.2	289.3±3.8	0.212	1.3±5.7	0.229	-0.369	2.243	0.139
Globulin-B	25.8±3.6	25.8±3.9	1.000	0.0±2.7	26.5±3.0	27.2±4.1	0.125	0.8±2.8	0.430	-0.279	1.266	0.265
ALB-B	45.6±2.6	45.7±3.0	0.942	0.0±2.4	44.8±2.9	44.8±2.5	1.000	0.0±2.9	0.266	0.012	0.002	0.962
BILT-B	0.5±0.2	0.5±0.3	0.780	0.0±0.2	0.5±0.4	0.5±0.4	0.828	0.0±0.2	0.571	0.006	0.001	0.982
Ca-B	9.4±0.4	9.4±0.4	0.702	0.0±0.4	9.4±0.4	9.4±0.4	0.704	0.0±0.4	0.800	-0.006	0.001	0.981
CR-B	0.8±0.1	0.8±0.2	0.119	0.0±0.1	0.8±0.2	0.8±0.2	0.526	0.0±0.1	0.938	0.152	0.381	0.539
GLU-B	93.2±9.4	95.4±13.3	0.303	1.8±10.2	89.9±9.2	89.7±10.3	0.899	-0.2±9.5	0.155	0.210	0.726	0.397
LDH-B	348.8±55.2	361.7±66.9	0.069	14.1±42.2	320.4±59.8	329.5±48.6	0.213	9.1±41.1	0.051	0.119	0.232	0.632
ALP-B	74.1±23.4	76.4±25.1	0.256	2.3±11.2	67.1±21.4	67.6±23.5	0.758	0.5±9.5	0.214	0.170	0.473	0.494
K-B	4.3±0.3	4.3±0.3	0.326	-0.1±0.3	4.3±0.3	4.3±0.4	0.597	-0.0±0.3	0.805	-0.111	0.206	0.652
PROT-T-B	71.3±3.4	71.3±4.5	0.956	0.0±3.2	71.2±4.1	71.9±5.0	0.372	0.7±4.4	0.906	-0.174	0.486	0.488
Na-B	139.7±1.7	139.2±2.2	0.323	-0.5±2.6	139.2±2.1	139.7±1.8	0.265	0.5±2.8	0.277	-0.373	2.294	0.135
AST-B	22.3±11.2	22.6±9.7	0.871	-0.3±10.8	17.6±4.3	19.1±8.4	0.212	1.4±6.4	0.030	-0.195	0.628	0.431
ALT-B	27.3±27.5	27.2±24.5	0.843	-0.8±23.1	17.0±7.4	19.3±14.5	0.199	2.4±10.4	0.044	-0.177	0.518	0.474
UREA-B	28.8±6.0	29.1±6.8	0.621	0.5±5.4	28.7±8.6	29.9±6.8	0.338	1.2±7.1	0.936	-0.115	0.216	0.643
CRP-B	3.4±4.1	4.5±6.0	0.012	1.1±2.4	2.5±3.5	2.9±3.6	0.352	0.4±2.3	0.376	0.298	1.467	0.23
Ferritin	154.0±101.1	140.8±90.3	0.345	-7.6±44.8	113.4±167.5	104.8±163.8	0.433	-8.6±58.4	0.254	0.020	0.006	0.938
URAC-B	4.9±1.4	5.0±1.3	0.376	0.1±0.8	4.8±1.4	4.9±1.5	0.237	0.1±0.6	0.843	-0.011	0.002	0.964

Data are presented as mean ± SD; Bold, significant after Bonferroni correction; * Cohen's d net effect size

Table 11: Adverse events during treatment period

AE	HBOT	Control
Barotrauma	4	3
Ear pain without barotrauma	1	0
Palpitation	3	1
Allergic rash	0	1
Headache	1	0
Emergency referral secondary to chest pain / epigastric pain	1	2
Fever	1	1
Urinary tract infection	0	2
Hospitalization due to herpes zoster infection	1	0
Cellulitis requiring antibiotic treatment	0	1
Pre-syncope	0	1
Hypertension	1	0
Pregnancy	0	1
Emotional distress with psychological intervention	0	1
**Total***	**13**	**14**

* P-value = 0.739

3 Supplementary Figures

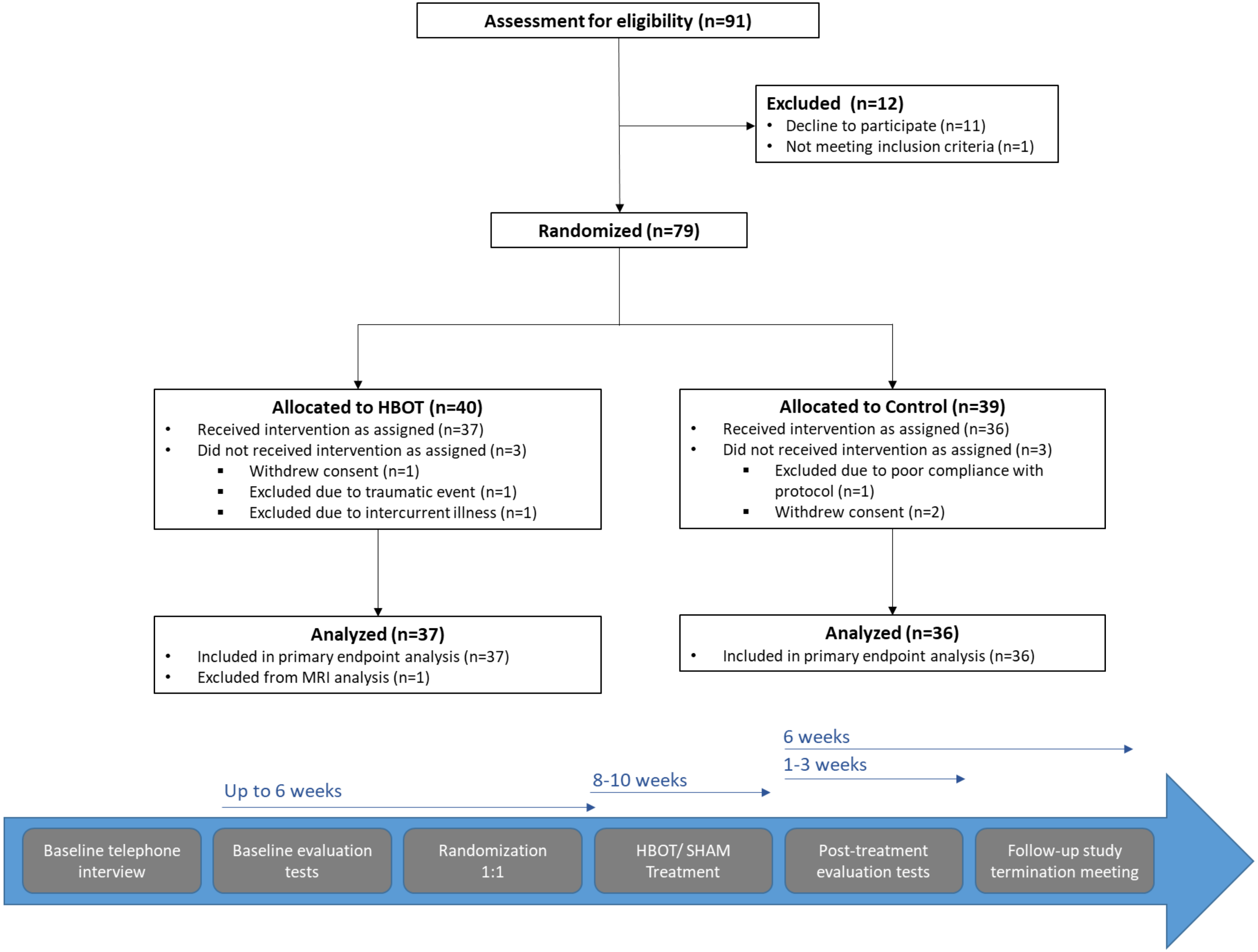

Figure 1. Study flowchart and timeline

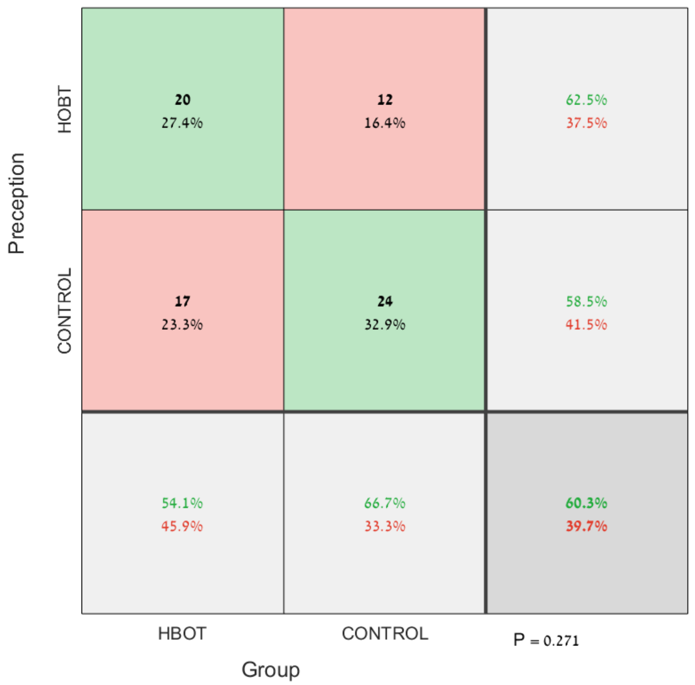

Figure 2. SHAM test results matrix. The green squares correspond to true perception, and the red squares represent false perception. The right column of the plot shows the percentages of the precision of the correct perception in each group separately. The overall correct perception rate is 60.3% (54.1% and 66.7% in the HBOT and CONTROL groups respectively).

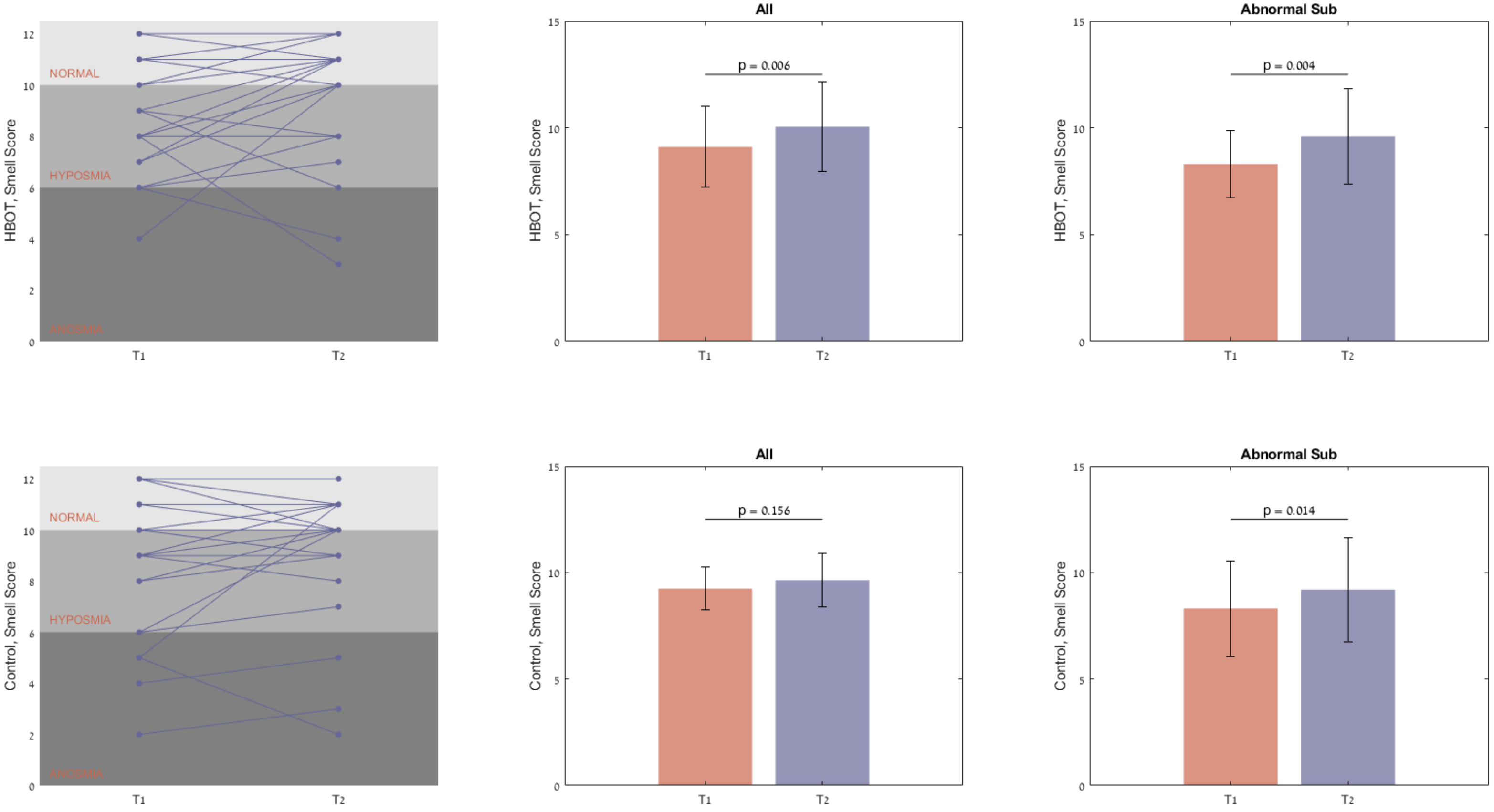

Figure 3. Smell total score changes in HBOT and control arms. Left plots, each line represents a patient's flow from baseline (T1) to post-intervention HBOT/Control (T2). Values are mean ± SD.

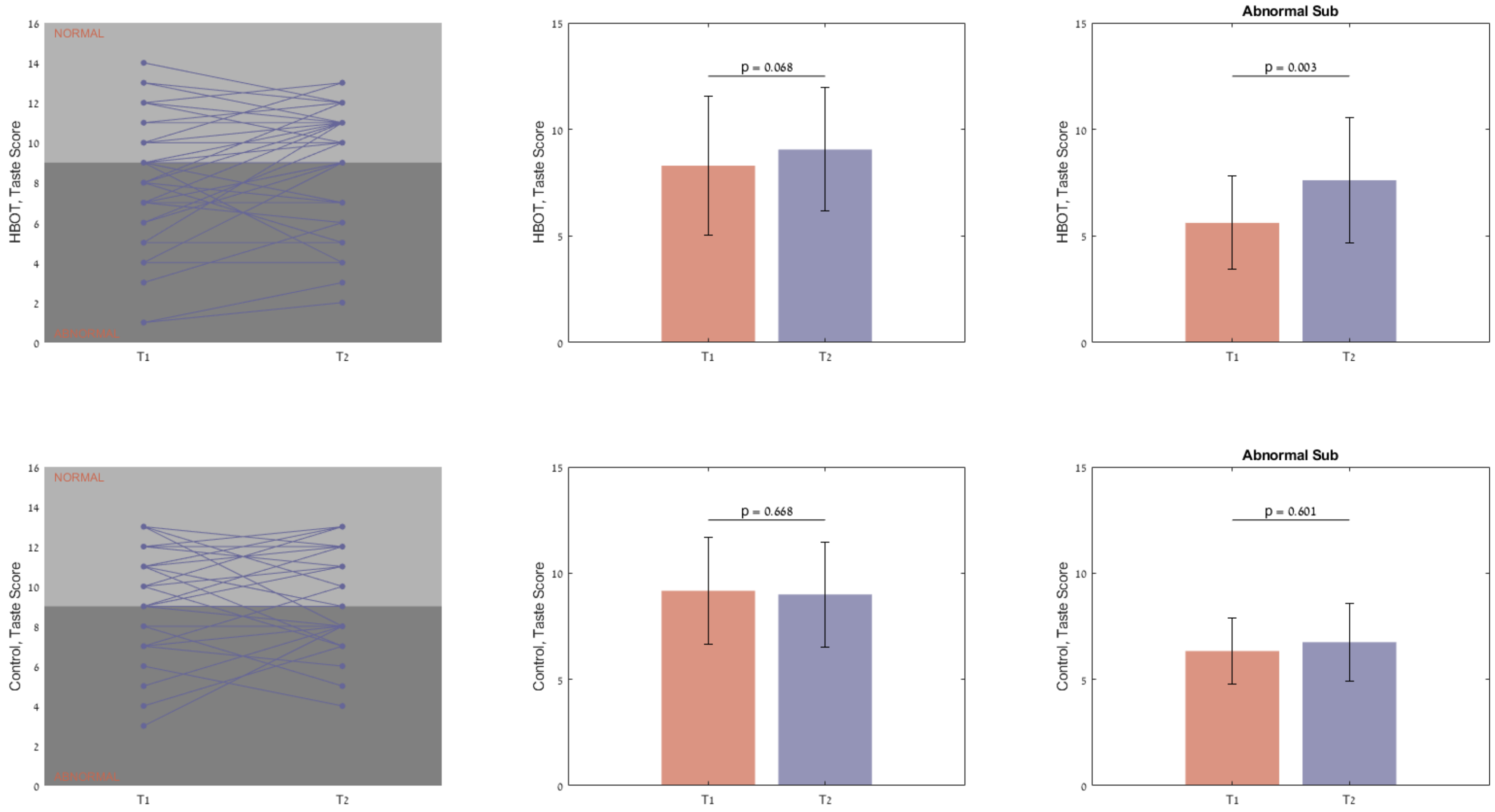

Figure 4. Taste total score changes in HBOT and control arms. Left plots, each line represents a patient's flow from baseline (T1) to post-intervention HBOT/Control (T2). Values are mean ± SD.
